# Supplementary figures and images for: Validation of the Emotiv EPOC EEG system for research quality auditory event-related potentials in children
Source: PeerJ. 2015 Apr 21;3:e907. doi: 10.7717/peerj.907 (PMC4411518; doi:10.7717/peerj.907)

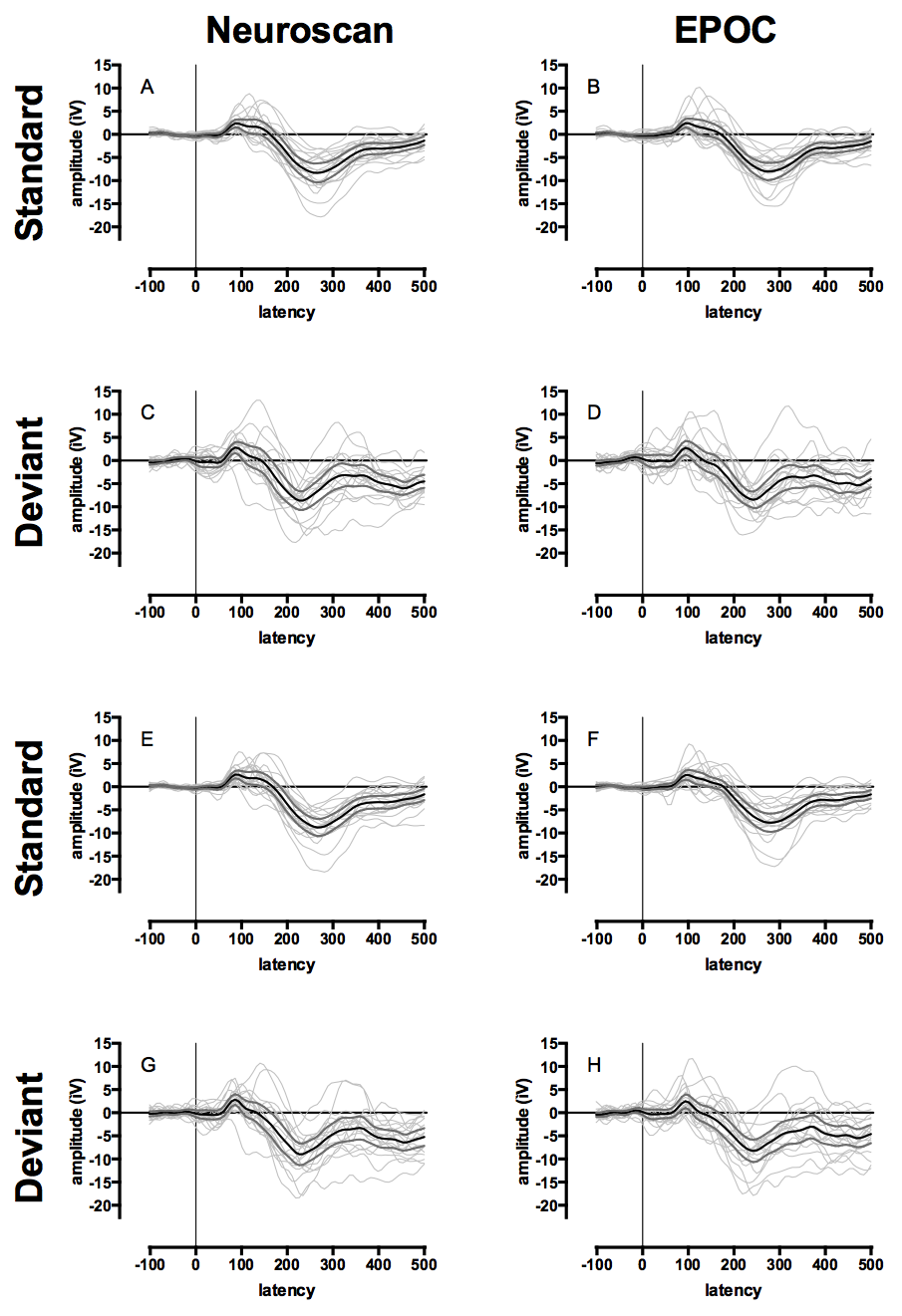

Supplement: Figure S1 — The auditory ERP waveforms of individuals (grey lines) at F3/AF3 and F4/AF4 for standard (panels A, B, E & F) and deviant stimuli (panels C, D, G, & H) in the passive condition for the Neuroscan (left; panels A, C, E, & G) and EPOC (right; panels B, D, F & H) systems. The black line represents mean and the bold grey are the 95% confidence intervals. [file peerj-03-907-s001.png]

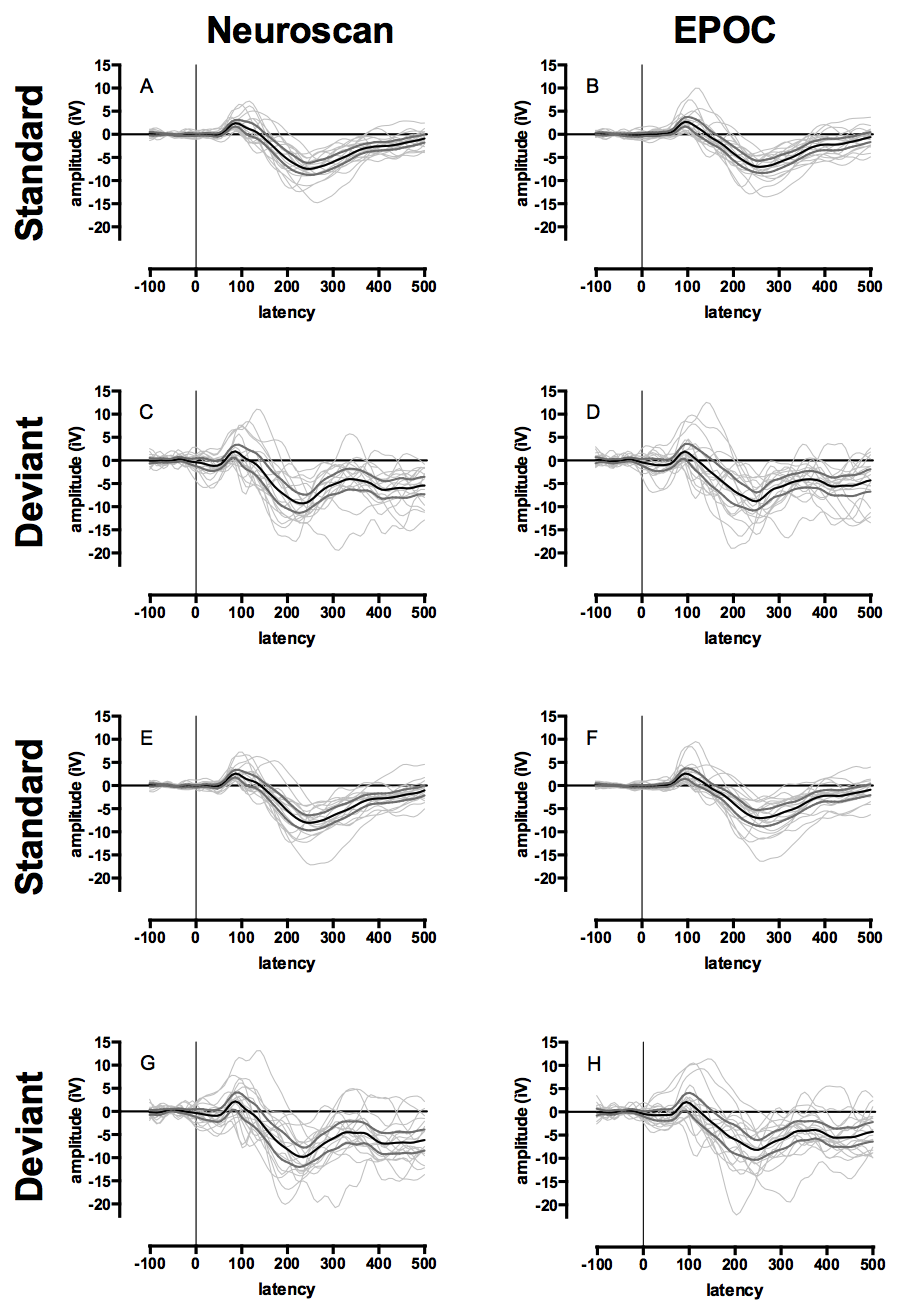

Supplement: Figure S2 — The auditory ERP waveforms of individuals (grey lines) at F3/AF3 and F4/AF4 for standard (panels A, B, E & F) and deviant stimuli (panels C, D, G, & H) in the passive condition for the Neuroscan (left; panels A, C, E, & G) and EPOC (right; panels B, D, F & H) systems. The black line represents mean and the bold grey are the 95% confidence intervals. [file peerj-03-907-s002.png]

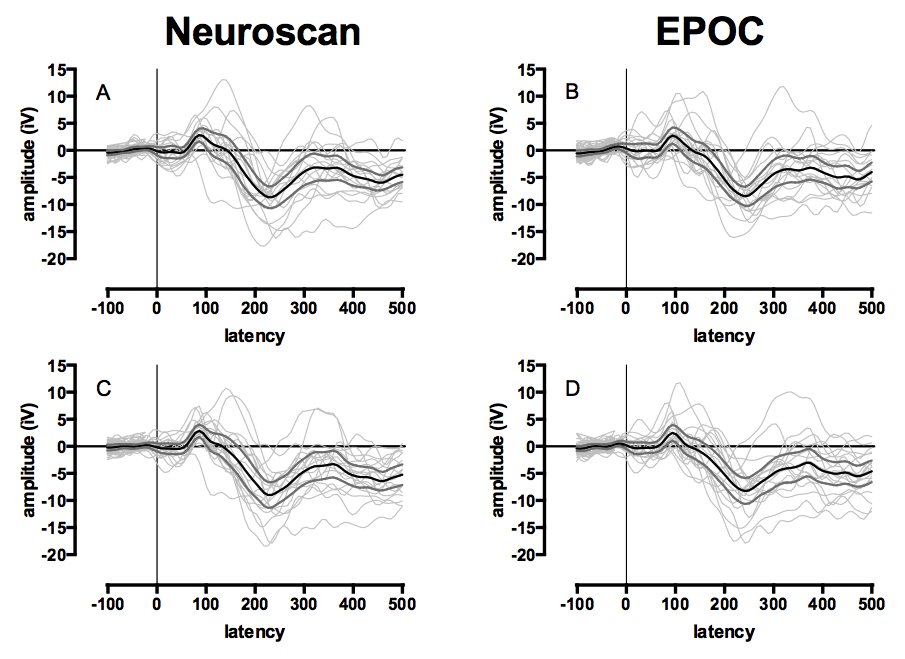

Supplement: Figure S3 — The MMN waveforms of individuals (grey lines) at F3/AF3 (panels A & B) and F4/AF4 (panels C & D) for standard and deviant stimuli in the passive condition for the Neuroscan (left; panels A & C) and EPOC (right; panels B & D) systems. The black line represents mean and the bold grey are the 95% confidence intervals. [file peerj-03-907-s003.png]
